# Visceral-to-subcutaneous fat ratio exhibits strongest association with early post-operative outcomes in patients undergoing surgery for advanced rectal cancer

**DOI:** 10.1007/s00384-022-04221-8

**Published:** 2022-07-28

**Authors:** Gabriele Bocca, Sotiris Mastoridis, Trevor Yeung, David R. C. James, Chris Cunningham

**Affiliations:** 1 Department of Colorectal Surgery, Oxford University Hospitals, Oxford, UK; 2 Nuffield Department of Surgical Sciences, University of Oxford, Oxford, UK

**Keywords:** Advanced rectal cancer, Body composition parameters, Surgical outcomes, Visceral-to-subcutaneous fat ratio, Prehabilitation

## Abstract

**Aim:**

Despite their promise as prognostic factors in colorectal cancer, anthropometric data are frequently contradictory or difficult to interpret, with single body-composition parameters often investigated in isolation or heterogeneous clinical cohorts used in analyses. We sought to assess a spectrum of body-composition parameters in a highly selected cohort with locally advanced rectal cancer in a bid to determine those with strongest prognostic potential in this specific setting.

**Materials/methods:**

Between 2014 and 2020, 78 individuals received neoadjuvant chemotherapy, or chemoradiotherapy, followed by radical surgery in the treatment of locally advanced rectal adenocarcinoma at Oxford University Hospitals Trust. Demographic, treatment-related, perioperative, and short-term outcomes data were assessed. Body-composition parameters included BMI, and those derived from pre-operative computed-tomography imaging: skeletal mass index (SMI), visceral fat area (VFA), subcutaneous fat area (SFA), perinephric fat area (PFA) visceral-to-subcutaneous fat ratio (V/S), sarcopenia, and sarcopenic obesity (SO).

**Results:**

Pre-operative body-composition parameters exhibited particularly strong correlation with post-operative outcomes, with VFA (*p* = 0.002), V/S (*p* = 0.019), SO (*p* = 0.012), and PFA (*p* = 0.0016) all associated with an increased length of hospital stay. Univariate and multivariate analyses demonstrated V/S to be the sole independent body-composition risk factor to be associated with an increased risk of developing Clavien–Dindo complications ≥ 2 (*p* = 0.033) as well as an increased length of stay (*p* = 0.005).

**Conclusion:**

Among patients with locally advanced rectal cancer, high visceral-to-subcutaneous fat ratio is the body-composition parameter most strongly associated with poor early post-operative outcomes. This should be considered in patient selection and prehabilitation protocols.

**What does this paper add to the literature?:**

Our study demonstrates that among body composition parameters, high visceral-to-subcutaneous fat ratio is strongly associated with increased risk of post-operative complications and increased length of stay in patients undergoing surgery for advanced rectal cancer.

## Introduction

Colorectal cancer (CRC) is the third most common neoplastic disease and accounts for 10.2% of all new cancer diagnoses. CRC is the second most common cause of death from cancer after lung neoplasms [1]. Combination therapy involving chemoradiotherapy (CRT) and total mesorectal excision (TME) surgery is currently considered the treatment of choice for patients with locally advanced rectal adenocarcinoma, threatened circumferential resection margin (CRM), or extramural venous invasion (EMVI) and/or tumour deposits [2, 3].

Rectal cancer surgery is potentially morbid and is associated with prolonged hospital stay in 17.7% of cases and a readmission rate of 14.0% [4]. Complications following rectal cancer surgery are associated with significant social and economic burden. The frequency of anastomotic leak following anterior resection is 10.9% and the cost of treating anastomotic leak is £17,220 per case [5].

Neoadjuvant treatment may have a deleterious impact on nutritional state and body composition and may therefore influence post-operative outcomes as well as disease-free survival. Abnormal body composition parameters may enable the detection of high-risk patients allowing them to be targeted for prehabilitation so that they can be optimized prior to surgery. Therefore, the time taken to complete neoadjuvant treatment could potentially be used to optimize a patient’s health prior to surgery.

Body composition, defined as the proportions and distribution of adipose tissue and skeletal muscle, is increasingly recognized to impact significantly on disease prognosis and treatment planning [6]. Several clinical populations, and in particular those with malignancy, have been shown to display adverse clinical outcomes in the presence of abnormal body composition features including, though not limited to, derangements in skeletal muscle mass, visceral adiposity, subcutaneous adiposity, and so on. Identifying patients with abnormal body composition can represent a clinical challenge. The availability of precise modalities such as dual-energy X-ray absorptiometry or air-displacement plethysmography, as well as the expertise to analyse such data, is limited. For this reason, clinicians and researchers are often limited to either crude measures such as body mass index (BMI) which do not inform on body composition and on the distribution of muscle and fat; or they employ indirect and relatively inaccurate measures such as bioimpedance. In contrast, computed tomography (CT) scans are performed relatively commonly and are widely available, and have been shown to be capable of provided accurate assessments of body composition. Typically, to aid in the ease and reproducibility of interpretation, body composition parameters including the visceral fat area (VFA), the subcutaneous fat area (SFA), the perinephric fat area (PNF), and the skeletal muscle index (SMI) can be retrieved from a single CT slice image [7, 8], using a specific bony landmark — most commonly the third lumbar vertebrae (L3) [9].

Changes in patients’ body composition (VFA, SFA, PNF, and SMI) during oncological treatments have a significant impact on both morbidity and long-term survival of patients affected by hepato-pancreatico-biliary, oesophagogastric, and colorectal malignancies [10–13]. Few studies have investigated the effects of neoadjuvant chemoradiotherapy (nCRT) and neoadjuvant chemotherapy (nCHT) on body composition and their association with postoperative outcome and survival in patients with advanced rectal cancer [14, 15]. We hypothesize that in this cohort of patients, body composition parameters may be affected by neoadjuvant treatments, and may be associated with outcomes following surgery.

Here we look at a highly selected group of patients with locally advanced rectal cancer who have undergone treatment followed by definitive surgery. We examine the following body composition parameters: body mass index (BMI), skeletal muscle index (SMI), visceral fat area (VFA), subcutaneous fat area (SFA), visceral fat area/subcutaneous fat area ratio (V/S ratio), and linear perinephric fat (PNF). In this study, we assess how these parameters are affected by neoadjuvant treatment, and assess their association with early surgical outcomes.

In this specific cohort of patients, we demonstrate for the first time that visceral fat area is associated with increased length of hospital stay, and visceral/subcutaneous fat ratio (V/S) is associated with both an increased risk of major complications and increased length of hospital stay.

## Methods

### Patients

Patients were identified and data were collected in a prospectively maintained database. All patients diagnosed with locally advanced rectal adenocarcinoma (cT2 + , N + , threatened CRM, EMVI + , R1 and/or recurrences after local treatment) who received neoadjuvant chemoradiotherapy (nCRT) or neoadjuvant chemotherapy (nCHT) combined with chemoradiotherapy (nCRT) followed by radical surgery over a period of six consecutive years (1st of January 2014–31st of January 2020) at Oxford University Hospital NHS Foundation Trust were included. Patients who received only neoadjuvant radiotherapy (nRT) or nCHT, patients who underwent lung or liver resections for metastatic disease before rectal surgery, and patients who had neoadjuvant treatment and had no surgery or had local excision only were excluded. All patients were discussed both at diagnosis and preoperatively at our local colorectal cancer Multidisciplinary Team meeting (MDT). Prospectively collected data included the following: age, gender, American Society of Anesthesiologists (ASA) score, smoking status, physical exercise, site of the malignancy, clinical stage American Joint Committee on Cancer (AJCC), TNM stage, neoadjuvant treatment regimens, surgical procedure, Clavien Dindo score (CD) complication, type of complication, length of stay, 30-day readmission, 30-day mortality, requirement of blood transfusions, haemoglobin (Hb), albumin, and C-reactive protein (CRP).

Body mass index (BMI), skeletal muscle index (SMI), visceral fat area (VFA), subcutaneous fat area (SFA), visceral fat area/subcutaneous fat area ratio (V/S ratio), and linear perinephric fat (PNF) were determined for each patient using computed tomography images taken (i) at the time of diagnosis (pre-neoadjuvant therapy) and (ii) prior to their resection procedure (post-neoadjuvant therapy) as described in detail below. Institutional approval was granted and the study was entered into the local audit register (IRID-6388).

### Body composition analyses

**Fig. 1:**
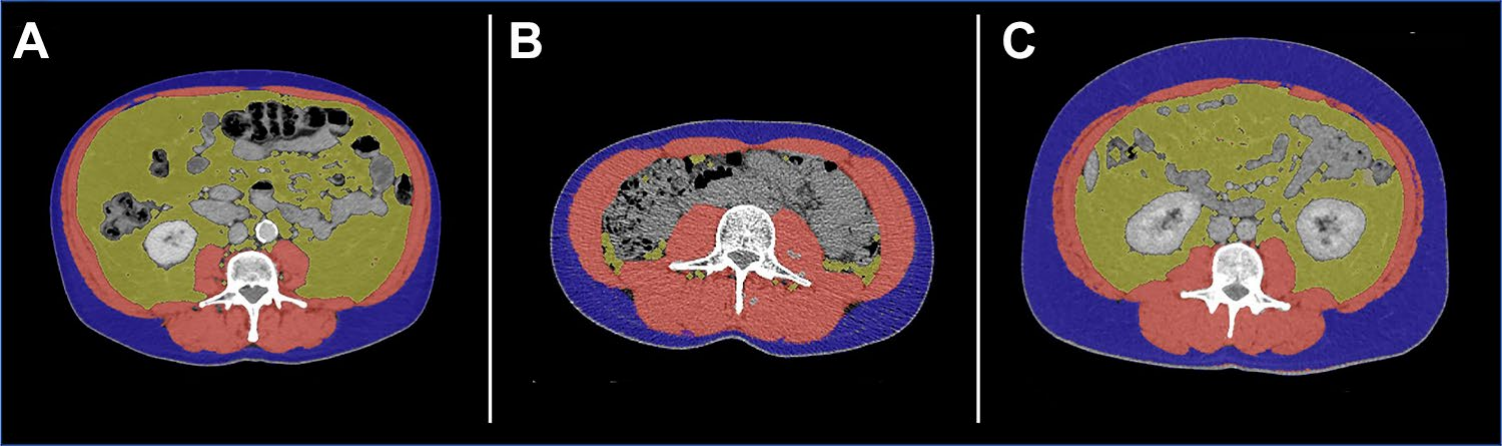
Representative CT scans assessed using CoreSlicer with the following body parameters highlighted: visceral fat (VFA, yellow), skeletal muscle (red), subcutaneous fat (SFA, blue). Examples of patients with **A** high VFA, low SFA; **B** low VFA, low SFA; **C** high VFA, high SFA

2.5-mm slice CT scan images at the level of the third lumbar vertebra (L3) including both transverse processes were selected to calculate patients’ skeletal muscle area (SMA), visceral fat area (VFA), and subcutaneous fat area (SFA)11. L3 slices have been demonstrated to accurately estimate the whole-body composition in both healthy [9] and cancer patients [7]. Parameters were calculated using the CoreSlicer.com web-based software package (v.1.0.0, Montreal, Quebec), as previously described and validated (Fig. 1) [16]. Indices including the CT images were anonymized, transferred in Digital Imaging and Communications in Medicine (DICOM) format, and analysed on standard desktop computers using CoreSlicer. Each CT image analysis was performed by three trained investigators independently to reduce the risk of investigator bias. The mean was then utilized. Investigators were blinded at the time of analysis with regard to patient identity, treatment modality, and outcomes. Interobserver reliability in the analysis of CT images was confirmed using Spearman’s rank correlation coefficient.

A number of indices were then derived from the above parameters [17, 18]. Skeletal muscle index was calculated using the mean skeletal muscle area normalized with the patient’s body surface area (cm^2^/m^2^). The V/S ratio was calculated using the mean VFA and SFA measurements [13]. Visceral obesity was defined as a V/S ratio of greater than 0.4 [19]. Sarcopenia was defined using gender-specific SMI cut-offs (< 38.5 cm^2^/m^2^ in women and < 52.4 cm^2^/m^2^ in men) [8, 20–22]. BMI was defined as the body mass divided by the square of the body height (kg/m^2^). In addition, linear perinephric fat (PNF) was derived as the shortest distance (mm) between the kidney and the abdominal wall as measured at the level of the renal veins [13, 23].

### Outcomes

Short-term post-operative outcomes include complications stratified for severity according to the Clavien–Dindo Classification, type of complication, 30 days’ readmission, 30-day mortality, and need for perioperative blood transfusion.

### Statistical analysis

Data are presented as mean ± standard deviation (SD) or median ± interquartile range (IQR) as indicated. Groups were compared using the chi-square test or Fischer’s exact for categorical variables, the two-way analysis of variance (ANOVA) or *t*-test for normally distributed data, and the nonparametric Kruskal–Wallis or Mann–Whitney *U* test for non-normally distributed variables. Bivariate correlation analyses were performed using Pearson’s test. For the test of potential risk factors associated with the outcomes, univariate analyses with clinically relevant parameters were performed. Variables with a value of *P* < 0.05 in the univariate analyses were included in the subsequent multivariate (logistic regression) analyses. All of the tests were two-sided and considered statistically significant when *P* values were less than 0.05. Statistical analyses were performed using the Statistical Package for the Social Sciences (SPSS) v26.0 (SPSS, Chicago, IL, USA), and GraphPad Prism v8.0.

## Results

A total of 78 patients were analysed in this study. Patient characteristics are shown in Table 1. Median age was 66 years and ranged from 31 to 90 years. Males accounted for 69.2% (*n* = 54), and females 30.8% (*n* = 24). Forty-four (56.4%) patients underwent abdominoperineal resection of the rectum (APER), 31 (39.7%) underwent low anterior resection, two (2.6%) underwent high anterior resection, and one (1.3%) underwent panproctocolectomy. All patients received neoadjuvant treatment. Fifty-six (71.8%) had chemoradiotherapy and 22 (28.2%) had chemotherapy, followed by chemoradiotherapy.

**Table 1:** Demographics of the study population

	No. of patients, N (%)
Age (years)	66 (31–90)
Sex ratio (M:F)	54:24
ASA fitness grade I–II III–IV	72 (92.3) 6 (7.7)
Surgical approach APER HAR LAR PPC	44 (56.4) 2 (2.6) 31 (39.7) 1 (1.3)
Comorbidity: Diabetes mellitus Hypertension Asthma/COPD Coronary artery disease	4 (5.1) 27 (34.6) 12 (15.4) 4 (5.1)
Smoking At diagnosis Ex-smokers	10 (12.8) 20 (25.6)
Pathological stage 0 I II III	12 (15.4) 8 (10.3) 23 (29.5) 35 (44.9)
Lymphovascular invasion No Yes	44 (56.4) 34 (43.6)
Neoadjuvant regimen CRT CHT + CRT	56 (71.8) 22 (28.2)

The body composition profiles calculated from CT analysis pre- and post-neoadjuvant therapy are shown in Table 2. There were no significant changes in any of the body composition profiles in our patient population after undergoing neoadjuvant therapy.

**Table 2:** Body composition parameters and profiles pre- and post-neoadjuvant therapy. Continuous variables were presented as means ± standard deviations (SD, normally distributed variables) or medians ± interquartile ranges (IQR, non-normally distributed variables). Continuous parameters pre- and post-neoadjuvant therapy were compared by paired *T* test or Wilcoxon matched-pairs signed rank test based on normality testing. Group comparisons involving dichotomous or categorical variables were performed using the chi-square test or Fisher’s exact test as appropriate

	Pre-neoadjuvant therapy	Post-neoadjuvant therapy	*P*
SMI, mean (SD)	50.9 (8.2)	50.8 (8.0)	ns
VFA, mean (SD)	179.9 (115.5)	177.7 (104.5)	ns
SFA, median (IQR)	163.6 (34.7)	163.0 (23.3)	ns
VFA/SFA ratio, median (IQR)	0.90 (0.42)	0.84 (0.26)	ns
PFA, median (IQR)	12.4 (4.5)	11.8 (3.9)	ns
BMI, mean (SD)	27.1 (4.3)	27.0 (3.5)	ns
Sarcopenia Yes, % (*n*) No, % (*n*)	36.8 (28) 63.2 (48)	36.8 (28) 63.2 (48)	ns
Sarcopenic obesity Yes, % (*n*) No, % (*n*)	4.8 (3) 95.2 (60)	9.2 (7) 90.8 (69)	ns
Visceral obesity Yes, % (*n*) No, % (*n*)	63.6 (49) 36.4 (28)	64.5 (49) 35.5 (27)	ns
Perinephric obesity Yes, % (*n*) No, % (*n*)	35.1 (27) 64.9 (50)	37.7 (29) 62.3 (50)	ns
BMI obesity Yes, % (*n*) No, % (*n*)	26.3 (15) 73.7 (42)	19.5 (15) 80.5 (62)	ns

Body composition parameters pre- and post-neoadjuvant therapy and their relationships with short-term outcomes following rectal cancer surgery are shown in Table 3. Ten individuals encountered complications of CD ≥ 3 including the following: anastomotic leak (4), SSI (2), major bleeding (2), compartment syndrome of lower limb (1), and small bowel obstruction (1). Twenty-three individuals encountered CD2 complications including the following: bowel complications (12), SSI (7), sepsis of unknown origin (2), wound complications (2), acute kidney injury or urinary tract infections (4), chest infection (1), infection of peripheral catheter (1).

**Table 3:** Body composition parameters pre- and post-neoadjuvant therapy and their relationships with short-term outcomes following rectal cancer surgery

	**Length of primary hospital stay (days)**				**Major complications within 30 days (CD ≥ 2)**						**30-day readmissions**					
	Pre-neoadjuvant treatment body composition analyses		Post-neoadjuvant treatment body composition analyses		Pre-neoadjuvant treatment body composition analyses			Post-neoadjuvant treatment body composition analyses			Pre-neoadjuvant treatment body composition analyses			Post-neoadjuvant treatment body composition analyses		
	**mean (SD)**	***P***	**mean (SD)**	***P***	**No**	**Yes**	***P***	**No**	**Yes**	***P***	**No**	**Yes**	***P***	**No**	**Yes**	***P***
**Overall**	10.00 (1.83)										68 (87.2)	10 (12.8)		70 (89.8)	8 (10.2)	
**Sarcopenia**																
**Yes, % (*n*)** **No, % (*n*)**	10.55 (1.90) 9.83 (1.82)	0.80	10.32 (1.84) 9.61 (1.84)	0.74	17 (60.7) 27 (56.3)	11 (39.3) 21 (43.7)	0.811	17 (60.7) 27 (56.3)	11 (39.3) 21 (43.7)	0.811	26 (92.9) 42 (87.5)	2 (7.1) 6 (12.5)	0.70	26 (92.9) 42 (87.5)	2 (7.1) 6 (12.5)	0.70
**Sarcopenic obesity**																
**Yes, % (*n*)** **No, % (*n*)**	23.13 (1.31) 9.80 (1.87)	*0.012	14.21 (1.70) 9.50 (1.83)	*0.05	2 (66.7) 33 (55.0)	1 (33.3) 27 (45.0)	> 0.9	6 (85.7) 38 (55.1)	1 (14.3) 31 (44.9)	0.228	3 (100.0) 57 (95.0)	0 3 (5.0)	> 0.99	7 (100.0) 66 (95.7)	0 3 (4.3)	> 0.99
**Visceral obesity**																
**Yes, % (*n*)** **No, % (*n*)**	11.65 (1.87) 7.72 (1.62)	**0.002	11.53 (1.87) 7.67 (1.61)	**0.004	30 (61.2) 15 (53.6)	19 (38.8) 13 (46.4)	0.637	29 (59.2) 15 (55.6)	20 (40.8) 12 (44.4)	0.811	43 (89.6) 25 (89.3)	5 (10.4) 3 (10.7)	> 0.99	45 (93.7) 22 (81.5)	3 (6.3) 5 (18.5)	0.13
**V/S Ratio (> 0.4)**																
**Yes, % (*n*)** **No, % (*n*)**	7.19 (1.57) 10.78 (1.86)	*0.019	8.14 (1.61) 10.29 (1.86)	0.29	9 (75.0) 35 (54.7)	3 (25.0) 29 (45.3)	0.222	8 (80.0) 36 (54.5)	2 (20.0) 30 (45.5)	0.177	12 (100.0) 61 (95.3)	0 3 (4.7)	> 0.99	10 (100.0) 63 (95.5)	0 3 (4.5)	> 0.99
**Perinephric obesity**																
**Yes, % (*n*)** **No, % (*n*)**	13.01 (1.91) 8.72 (1.73)	**0.0016	11.58 (2.00) 9.25 (1.71)	0.08	16 (59.3) 29 (58.0)	11 (40.7) 21 (42.0)	> 0.9	18 (62.1) 27 (56.3)	11 (37.9) 21 (43.7)	0.642	24 (88.9) 45 (90.0)	3 (11.1) 5 (10.0)	> 0.99	27 (93.1) 42 (87.5)	2 (6.9) 6 (12.5)	0.70
**BMI obesity**																
**Yes, % (*n*)** **No, % (*n*)**	11.15 (1.83) 9.56 (1.97)	0.14	11.40 (1.82) 9.64 (1.86)	0.11	9 (60.0) 23 (54.8)	6 (40.0) 19 (45.2)	0.771	10 (66.7) 35 (56.5)	5 (33.3) 27 (43.5)	0.567	15 (100.0) 38 (90.5)	0 4 (9.5)	0.56	14 (93.3) 57 (91.9)	1 (6.7) 5 (8.1)	> 0.99

For body composition parameters assessed pre-neoadjuvant therapy, VFA (*p* = 0.002), V/S ratio (*p* = 0.019), sarcopenic obesity (*p* = 0.012), and perinephric obesity (*p* = 0.0016) were all associated with an increased length of hospital stay. Among parameters assessed post-neoadjuvant therapy, VFA (*p* = 0.004) and sarcopenic obesity (*p* = 0.05) were significantly associated with increased length of hospital stay. Overall, patients that exhibited high VFA pre-neoadjuvant treatment had a mean length of hospital stay of 11.65 days compared to 7.72 days for those patients that exhibited low VFA.

To explore the relationship between body parameters pre-neoadjuvant therapy and outcomes, we performed univariate and multivariate regression analyses (Table 4). We found V/S ratio to be the sole body composition parameter to be associated with an increased risk of developing Clavien–Dindo complications ≥ 2 on univariate analysis (*p* = 0.033). V/S ratio was also associated with an increased length of stay (*p* < 0.0001), as were VFA (*p* < 0.0001) and PNF (*p* = 0.05). Multivariate analysis incorporating these parameters demonstrated V/S alone to be an independent risk factor for increased length of stay (*p* = 0.005).

**Table 4:** Univariate and multivariate linear and logistic regression analyses of body parameters pre-neoadjuvant treatment in relation to complications and length of stay. To model the relationship between pre-operative patient variables including body composition parameters, with measures of early post-operative outcomes, univariate analyses with clinically relevant parameters were performed. Variables with a value of *P* < 0.10 in the univariate analyses were included in the subsequent multivariate (logistic regression) analyses

Factors	CD ≥ 2	30-day readmission	LOS	
	**Univariate analysis** **(*P*)**	**Univariate analysis** **(*P*)**	**Univariate analysis** **(*P*)**	**Multivariate analysis** **(*P*)**
**Age**	0.987	0.404	0.148	
**Gender (M:F)**	0.287	0.263	0.107	
**Stage**				
** 0–I** ** II–III**	0.952	0.350	0.418	
**ASA**				
** I–II** ** III–IV**	0.144	0.977	0.773	
**Neoadjuvant regimen**				
** CHT**	0.875	0.318	0.317	
** CHT + CRT**				
**Albumin**	0.438	*0.020	0.943	
**Hb**	0.712	0.682	0.645	
**SMI**	0.993	0.448	0.876	
**BMI**	0.214	0.549	0.351	
**VFA**	0.052	0.456	*** < 0.0001	0.166
**SFA**	0.546	0.280	0.855	
**PNF**	0.094	0.864	**0.050**	0.189
**V/S ratio**	*0.033	0.822	*** < 0.0001	**0.005
**Sarcopenia**				
** Yes, % (*n*)** ** No, % (*n*)**	0.560	0.468	0.710	
**Sarcopenic obesity**				
** Yes, % (*n*)** ** No, % (*n*)**	0.966	> 0.9	0.372	

Analyses of short-term outcomes involving group comparisons of continuous non-parametric variables were performed by Mann–Whitney *U* test and Kruskal–Wallis *H* test. Group comparisons involving dichotomous or categorical variables were performed using the chi-square test or Fisher’s exact test as appropriate.

## Discussion

This study represents the largest single-centre analysis focusing specifically on a cohort of patients with locally advanced rectal cancer who have had neoadjuvant chemoradiotherapy prior to surgery, and which compares a breadth of body composition parameters on specific outcomes following surgery. We find that pre-neoadjuvant visceral-to-subcutaneous fat (V/S) ratio is strongly associated with increased risk of major complications and increased length of hospital stay. We also show that pre-neoadjuvant VFA is also strongly associated with increased length of stay. BMI was not associated with poorer outcomes in our cohort of patients. This is in keeping with other studies, where VFA and V/S ratio have been shown to be a better marker for metabolic X syndrome and poorer surgical outcomes, as opposed to BMI, which is a much cruder index of general adiposity and takes no account of fat distribution, nor ratio of fat to muscle mass.

Visceral adiposity is known to be associated with chronic, low-grade inflammation, and a higher risk of cardiovascular outcomes [24, 25] Animal models demonstrate visceral adipocytes as a source of inflammation and insulin resistance with the capacity to release adipokines and chemokines associated with metabolic syndrome and correlating with adverse cardiometabolic profiles and tumour outcomes [26–28]. Compared with visceral adiposity, subcutaneous fat contains lower numbers of inflammatory and immune cells and is considered to be less active in driving metabolic syndrome [29]. Another reason which may impact on why those with visceral obesity may have poorer outcomes is a practical one, namely the surgical difficulty associated with the surgeon having deeper and poorer views of the surgical field both laparoscopically and open, as well as fragile tissues which tend to bleed more readily among this patient cohort.

We found that body composition parameters pre-neoadjuvant therapy were more strongly associated with increased length of hospital stay than the same body parameters measured post neoadjuvant treatment. At diagnosis, VFA, V/S ratio, sarcopenic obesity, and perinephric obesity were all associated with an increased length of hospital stay. After neoadjuvant treatment, only VFA was associated with increased length of stay. Patients undergoing neoadjuvant treatment are susceptible to body composition changes, as chemoradiotherapy can lead to nausea, loss of appetite, diarrhoea, and subsequent weight loss. We examined the change in body composition parameters between diagnosis and pre-operative state and found that these changes were not associated with increased length of stay or the development of major complications.

Whilst other studies have demonstrated an association between sarcopenia and increased complication rates, we did not see this association in our patient population. This might be due to our study involving a highly selected group of patients who have undergone surgery for locally advanced rectal cancer following neoadjuvant chemoradiotherapy. One of the limitations of our study is that we examine short-term outcomes following surgery. Future studies are indicated to examine whether VFA and V/S ratios have a significant effect on long-term disease-free survival.

## Conclusion

Visceral/subcutaneous (V/S) fat ratio serves as the strongest independent risk factor among body composition parameters for increased length of hospital stay and post-operative complications in patients with locally advanced rectal cancer undergoing neoadjuvant chemoradiotherapy.
